# Piezoelectric Versus Conventional Osteotomy Instruments in Bimaxillary Orthognathic Surgery: A Narrative Review of Surgical Efficiency, Safety, and Clinical Outcomes

**DOI:** 10.3390/jcm15207818

**Published:** 2026-10-10

**Authors:** Renato Isufi, Aurora Isufi, Ramazan Isufi, Majeed Rana

**Affiliations:** 1 University Dental Clinic, University of Medicine, 1011 Tirana, Albania; 2 Isufi Academy, 1011 Tirana, Albania; aurora_3131@hotmail.com; 3 Faculty of Dental Medicine, University of Medicine, 1005 Tirana, Albania; prof.isufi_r@hotmail.com; 4 Department of Oral, Maxillofacial and Facial Plastic Surgery, University Hospital Düsseldorf, Heinrich Heine University, 40225 Düsseldorf, Germany; rana@med.uni-duesseldorf.de

**Keywords:** orthognathic surgery, bimaxillary surgery, piezosurgery, piezoelectric osteotomy, Le Fort I osteotomy, bilateral sagittal split osteotomy, blood loss, neurosensory disturbance

## Abstract

Instrument selection in bimaxillary orthognathic surgery must balance cutting efficiency, measured blood loss, and injury to adjacent structures. This narrative review distinguishes the evidence from combined Le Fort I and bilateral sagittal split osteotomy (BSSO) procedures from the evidence concerning individual osteotomies. A targeted PubMed update through 24 September 2026, supplemented by reference tracing and publisher and repository records, informed a study-level appraisal. Several comparative studies and meta-analyses report lower measured blood loss with piezoelectric instrumentation, but a non-significant pooled comparison and methodological limitations prevent a uniform estimate of benefit. A reduction in transfusion or major vascular injury has not been established. Neurosensory findings are favorable in some studies but inconsistent across tests and follow-up intervals; time to functional sensory recovery, degree of recovered function, and persistent disturbance are distinct outcomes. Operative-time results also conflict, partly because studies measure different intervals. Operator experience is a possible contributor rather than a demonstrated explanation. Evidence for clinically important improvements in pain, swelling, bone healing, or skeletal stability remains limited. Technical reports support the feasibility of selective or hybrid instrumentation, but comparative superiority of a hybrid workflow has not been established. Current evidence supports cautious, procedure-specific interpretation and further comparative trials rather than a universal replacement of conventional instruments.

## 1. Introduction

Bimaxillary orthognathic surgery usually combines Le Fort I maxillary osteotomy with BSSO to correct maxillary and mandibular deformities. Virtual planning and rigid fixation improve control of skeletal positioning, but osteotomy accuracy, thermal injury, and preservation of nearby mucosa, vessels, and nerves remain relevant to clinical outcomes [1,2,3,4]. Conventional instrumentation includes reciprocating or oscillating saws, rotary burs, osteotomes, and chisels. These instruments differ in cutting action and should not be treated as a single standardized comparator.

Piezoelectric osteotomy, also termed piezosurgery or piezoelectric bone surgery in this review, uses ultrasonic insert motion to cut mineralized tissue with relative soft-tissue selectivity [5,6,7,8]. This technical property provides a rationale for work near vulnerable anatomy; it does not itself demonstrate fewer clinical nerve injuries. Ultrasonic bone scalpels and curettes are identified separately when a study evaluates them as a distinct device category, because geometry, power, and complete surgical workflows may differ.

Existing systematic reviews address pooled complications or sensory outcomes, while a recent scoping review maps neurosensory evidence [9,10,11,12,13,14,15,16]. The unresolved question is how those findings apply to the different steps of a combined operation. This review therefore separates bimaxillary outcomes from maxillary and mandibular endpoints, compares the actual definitions used in key studies, and distinguishes demonstrated clinical findings from anatomical reasoning. The proposed role of hybrid instrumentation is considered as a research question, not as an established superior treatment.

## 2. Literature Search and Appraisal Approach

This is a narrative review, not a systematic review or a new meta-analysis. Its reporting is informed by SANRA [17]. The original search history was not retained. To make the revision auditable, a targeted literature update and source verification exercise was performed on 24 September 2026. The update should not be interpreted as a reconstruction of the original search or as exhaustive identification of all relevant publications.

PubMed was searched using: (piezosurgery OR piezoelectric OR piezoosteotomy OR piezotome OR ultrasonic) AND (orthognathic OR bimaxillary OR “Le Fort” OR “sagittal split”). The publication-date range was 1 January 1900 to 24 September 2026; no language or study-design filter was applied at retrieval. This search returned 161 records. An initial narrower query omitting piezotome and ultrasonic returned 119 records. Titles were inspected for relevance, and abstracts of relevant comparative studies and reviews were examined. Additional sources were the reference lists of relevant reviews, PubMed Central, journal pages on Elsevier/ScienceDirect, Frontiers, MDPI and Annali Italiani di Chirurgia, and the institutional repositories of the Universities of Milan and Florence. These were supplementary article retrieval sources, not independent comprehensive database searches. Scopus, Embase, and Web of Science were not independently searched for this revision.

Selection was purposive: human comparative studies of combined bimaxillary surgery, Le Fort I osteotomy, or sagittal split osteotomy were used for clinical comparisons, with particular attention to studies underlying the existing manuscript and the reviewer concerns. Randomized and prospective controlled evidence was given greater interpretive weight when allocation, assessment, and follow-up were adequately described. Retrospective and historical-control studies were retained with explicit limitations. Reviews were used to contextualize the primary studies and their overlap. Technical reports and preclinical studies were included only for feasibility or mechanisms; third molar surgery was treated as indirect evidence. Conference abstracts, dissertations, trial registries, and other gray literature pieces were not systematically searched. Detailed extraction was limited to English-language reports or English abstracts that could be accessed; full-text availability was not an exclusion criterion.

Numerical findings were checked against original publication records and accessible full texts, rather than inferred from article titles. When only an abstract was accessible, unreported allocation, blinding, supplementary instruments, measurement scales, or protocols were designated as not verifiable from the accessible report. This does not mean that the full publication omitted them. No independent duplicate screening, formal risk-of-bias scoring, GRADE assessment, or new pooling was undertaken. Instead, the appraisal describes design, comparator, unit of analysis, outcome definition, follow-up, and specific threats to interpretation. Incomplete full-text access and non-systematic selection limit certainty. A non-significant difference is not treated as equivalence, and multiple reviews containing the same studies are not counted as independent confirmations.

## 3. Technical Principles and Procedural Context

Figure 1 provides an anatomical overview of Le Fort I osteotomy and BSSO. The maxillary osteotomy is related to the nasal lining, tooth roots and posterior palatine vessels, whereas the mandibular split is related to the inferior alveolar neurovascular bundle. These relationships explain why tissue protection and controlled cutting remain important regardless of the instrument used [1].

### 3.1. Cutting Mechanisms

A piezoelectric insert oscillates with a small amplitude. Cutting performance depends on insert geometry, power, pressure, irrigation, cortical thickness, and access [6,7,8,18,19,20]. Irrigant cavitation has been described as a mechanism that may improve visualization, but its independent contribution to clinical blood loss or complications has not been established. Excessive pressure or prolonged contact may impair cutting and generate heat. Relative tissue selectivity does not remove the need for soft-tissue protection and controlled advancement.

Saws provide rapid cortical cutting, burs permit localized bone removal, and osteotomes or separators complete mobilization or splitting. These are technical characteristics and practical considerations rather than proof of comparative clinical benefit. Importantly, a piezoelectric study group may still receive conventional instruments. In Landes et al. [21], auxiliary chisels were used for the septum and lateral nasal walls, for one third of pterygoid processes, and an auxiliary saw was required in 15% of mandibular osteotomies. Such comparisons evaluate a predominantly piezoelectric workflow, not the exclusive use of one device.

### 3.2. Evidence from Combined Bimaxillary Operations

The principal direct comparisons include the Landes series, the within-patient studies of Spinelli and Akbar, the trial reported by Bertossi, the longitudinal study of Rossi, and the study of Cascino [21,22,23,24,25,26,27]. Their designs and endpoints differ substantially (Table 1). Patient counts cannot be replaced by numbers of operated sides or osteotomies. Within-patient comparisons control many systemic factors but require paired analysis and may not cleanly separate whole-operation outcomes such as blood loss, medication use, or length of stay.

Cascino et al. describe a retrospective study in the abstract methods, despite “randomized” in the title and the indexing label [27]. The study is therefore characterized here as a retrospective comparison with unresolved allocation details, rather than assumed to be a randomized trial. The two 2008 Landes reports come from the same research group and related clinical experience [21,22]; independence of their cohorts should not be assumed.

### 3.3. Le Fort I Osteotomy

The nasal mucosa, descending palatine vessels, pterygomaxillary junction, and posterior maxillary structures make controlled depth and visualization relevant during Le Fort I osteotomy. Major vascular complications are recognized, but technical precision should not be equated with a proven reduction in those rare events [32,33]. Reports of nasal mucosal detachment and multipiece osteotomies demonstrate feasibility [34,35].

Demirbas et al. compared an ultrasonic BoneScalpel, a piezoelectric surgical device, and conventional instrumentation in 34 patients [30]. The BoneScalpel had shorter cutting and procedure times and lower blood loss; those findings must not be reassigned to the separate piezoelectric group. Bilge et al. studied pterygomaxillary separation patterns in 48 patients [36]. More recent comparisons of a palatal piezoelectric approach and an ultrasonic bone scalpel also report favorable radiographic separation patterns [37,38]. These are procedure- or step-specific findings. They do not establish fewer transfusions or better long-term skeletal stability after an entire bimaxillary operation.

### 3.4. Bilateral Sagittal Split Osteotomy

BSSO combines cortical cutting, splitting, nerve handling, repositioning, and fixation. The mandibular canal, lingual ramus, and posterior border constrain access. Traction, compression, nerve entrapment, mandibular movement, and patient characteristics may affect sensation independently of the cutting instrument [39,40,41,42,43,44,45]. Studies focused on BSSO are directly relevant to mandibular sensory function, but their blood-loss and time endpoints cannot automatically be extrapolated to a complete two-jaw operation. Detailed sensory comparisons are presented once, in Section 4.3 and Table 2.

## 4. Comparative Clinical Outcomes

### 4.1. Intraoperative Blood Loss

Several studies report lower measured blood loss with piezoelectric instrumentation, but not all comparisons are statistically significant. In the Landes reports, bimaxillary blood loss was approximately 541 ± 150 versus 773 ± 344 mL (*p* = 0.001) and 537 ± 208 versus 772 ± 338 mL (*p* = 0.0001), respectively [21,22]. These are historical-control comparisons with potential cohort overlap. Spinelli reported a 25% reduction (*p* = 0.0367), whereas Akbar’s blood-loss comparison was non-significant (*p* = 0.091) [23,26]. Shirota found no independent blood-loss advantage in a retrospective BSSO comparison [29].

The review by Pagotto et al. included eight controlled studies overall and reported a pooled mean blood-loss difference of −128 mL (*p* < 0.001) [14]. The overall number of included studies is not necessarily the number contributing to this endpoint; that denominator and the endpoint-specific confidence interval and heterogeneity could not be verified from the accessible abstract. Alrefai et al. reported a mean difference of −140.4 mL, but the comparison was non-significant (*p* = 0.29) [15]. Their favorable concluding wording therefore requires qualification.

Ahmadi et al. reported six studies, 144 participants and 252 procedures, with a weighted mean difference of −81.73 mL (95% CI −97.30 to −66.16; *p* < 0.001; I^2^ = 51.83%) [9]. Their table mixes patients, sides and procedures; no explicit paired-correlation adjustment is described. Source verification also identified extraction concerns: Sato’s original report describes 22 patients and blood loss in grams, whereas the review tabulates 20 patients and milliliters [31]. Consequently, the pooled precision cannot be fully assessed and the estimate is reported with reservations, not endorsed as a fixed clinical effect. The review used JBI appraisal but did not report a GRADE certainty rating.

Clinical importance depends on baseline loss and hemoglobin, the operation performed, anesthetic management, duration, irrigation subtraction, hemostatic measures, and transfusion practice. A difference near 80–140 mL may have different implications at low versus high baseline bleeding. Better visibility is plausible, but the cited mean differences alone do not demonstrate fewer transfusions, major vascular injuries, or clinically important improvements in recovery. No universal transfusion benefit or risk reduction can be estimated from these data.

### 4.2. Operative Time

Three intervals must be distinguished: device-specific cutting time, procedure time from incision to osteotomy or split completion, and total operative duration. Operating-room time also includes activities outside the operative interval. These outcomes are not interchangeable. Table 1 reports the available absolute values and their scope.

Köhnke et al. measured the interval from incision to complete mandibular splitting for each side in 50 patients (100 procedures). Piezoelectric surgery was faster: 17 ± 6 versus 25 ± 9 min (*p* < 0.001) [28]. Both specialists had at least one year of piezosurgery experience, but there was no randomized comparison of experience levels; experience cannot be claimed as the cause of the difference. Bertossi reported shorter osteotomy intervals for both mandibular and maxillary cutting [24]. Conversely, Spinelli reported 35% longer procedure duration (*p* = 0.0018), and Rossi reported longer total operation duration with piezosurgery [23,25]. The 63 min piezosurgery value reported by Akbar is not paired with an absolute saw time or a sufficiently precise interval definition in the accessible abstract [26]; it is not treated as a comparable total-operation estimate.

Landes reported no significant difference in total bimaxillary duration, and Cascino described unchanged intraoperative time [21,22,27]. Jenkins and Langford also found nearly identical operation duration in sequential cohorts [49]. Pagotto’s pooled comparison found no operative-time difference, while Silva and Alrefai described longer times with ultrasonic or piezoelectric instrumentation [11,14,15]. Device design, access, supplementary instruments, experience, and outcome definition are plausible contributors to heterogeneity. Their separate causal effects remain unestablished.

### 4.3. Neurosensory Outcomes

Early sensory disturbance, time to functional sensory recovery, degree of recovered function, and persistent impairment are distinct endpoints. Studies use subjective reports, light touch, two-point discrimination, pin-prick, Semmes–Weinstein monofilaments, or composite recovery scales [41,42,43,44]. A lower threshold on one test does not necessarily imply earlier functional recovery or less persistent disability. Table 2 aligns the principal studies by test and follow-up.

Bertossi reported retained inferior alveolar nerve sensation at six months in 98.2% versus 92.7% of the groups [24]. These proportions should not be treated as a precise absolute benefit without a verified denominator definition, confidence interval, and comparable assessment. In contrast, Köhnke found no difference in sensory disturbance or recovery through 12 months [28]. Monnazzi’s prospective split-mouth study, with blinded patients and examiner and Semmes–Weinstein testing, also found no significant instrument-related difference through six months [46]. Shirota’s retrospective BSSO study found greater three-month sensory disturbance with piezosurgery (*p* = 0.008), using a different comparator workflow and an adjusted analysis [29].

Sobol et al. followed 20 patients (40 sagittal split osteotomies) using the UK Medical Research Council functional sensory recovery scale [47]. Median recovery time was 94.5 versus 101.5 days (*p* = 0.20); all patients reached functional recovery within one year. Higher sensory scores at recovery favored piezoelectric sites (reported hazard ratio 2.3, 95% CI 1.1–4.9; *p* = 0.04). The latter finding concerns the degree of recovered function and does not establish faster recovery. Koba and Jenkins reported favorable sensory findings in retrospective comparisons, but residual confounding remains possible [48,49].

Review-level conclusions are discordant (Table 3). Rude et al. found only five eligible controlled studies, all at moderate or high risk of bias, and did not pool them [12]. Abd-ElHady et al. included six studies and 284 participants; the three-month risk ratio was 0.39 (95% CI 0.09–1.75; *p* = 0.22), which is compatible with considerable uncertainty [13]. Alrefai’s three- and six-month comparisons were also non-significant (*p* = 0.11 and *p* = 0.50), despite lower descriptive disturbance proportions in piezoelectric groups [15]. A scoping review documents heterogeneous recovery patterns but cannot establish a reliable pooled effect [16].

Split-mouth designs reduce between-patient confounding, while blinding limits measurement bias, but small samples remain imprecise and bilateral observations are correlated. Retrospective comparisons may differ in canal anatomy, movement magnitude, nerve manipulation, fixation, and perioperative care. Available reports do not allow a reliable instrument-by-technique analysis for Hunsuck versus Epker modifications. They also do not establish whether any benefit is independent of movement or surgeon experience. Accordingly, the evidence does not support a precise absolute reduction in persistent sensory disturbance or a guarantee of nerve preservation.

### 4.4. Pain Swelling and Postoperative Recovery

Rossi compared 14 piezosurgery patients with 11 saw patients and found significant differences in pain on day three (*p* = 0.035) and swelling on day 30 (*p* = 0.045) [25]. The accessible abstract does not provide the group values, measurement-scale details, or a clinically important difference threshold. It therefore supports specific time-point findings, not a quantifiable patient-noticeable benefit. Spinelli reported less swelling and hematoma, the small within-patient sample and possible spread of edema across the midline limit attribution [23]. A small additional split-mouth study reported greater swelling on the piezoelectric side, reinforcing that morbidity results are not uniformly favorable [50].

Cascino described less analgesic use and discharge on postoperative day two in the piezoelectric group [27]. The medication doses, rescue thresholds, steroid regimen, comparator discharge timing, and discharge criteria could not be verified from the accessible report. These observations cannot establish a protocol-independent reduction in analgesic requirements or length of stay. Pain and edema comparisons should account for operative duration, tissue handling, analgesia, steroids, and assessment methods. Third molar evidence remains indirect and does not increase certainty for bimaxillary operations [51].

### 4.5. Precision Healing and Complications

Narrow cuts and controlled insert movement are technical properties, supported by device descriptions and feasibility reports [18,19,20,34,35]. They are not validated surrogates for union, relapse, or patient-reported recovery. Esteves et al. found broadly similar preclinical healing dynamics with limited time-point differences [52]. Comparative clinical evidence remains insufficient to favor either instrument for union, consolidation, or long-term skeletal stability.

Shirota did not demonstrate a reduction in blood loss or neurosensory disturbance with piezosurgery [29]. For unfavorable splits, Salzano et al. reviewed 1120 BSSROs and observed five bad fractures across burr and saw groups, with no statistically significant association with instrument choice [53]. Very few events limit any inference that piezosurgery eliminates this risk. Maxillary complication reviews likewise identify insufficient high-quality comparative evidence [10].

Cross-specialty adverse-event surveillance describes possible device hazards, including malfunction and tissue injury [54]. Such surveillance lacks a controlled bimaxillary denominator and cannot provide comparative complication rates for this operation. Conventional and piezoelectric workflows both require anatomical protection, appropriate depth, irrigation, and safe handling of supplementary instruments.

## 5. Interpretation and Practical Implications

The outcome summary in Table 4 separates clinical comparative evidence from technical properties and practical assumptions. Greater weight should be given to clearly defined controlled comparisons and blinded sensory testing than to descriptive advantages in uncontrolled series. Nevertheless, a randomized label alone does not establish reliable allocation concealment or complete follow-up. Bertossi’s larger trial is directly relevant to bimaxillary surgery, but the accessible abstract does not permit the full appraisal of these features. Köhnke and Monnazzi provide useful within-patient comparisons with explicit assessment methods; their null sensory results must remain visible alongside positive studies. Cascino, Landes, Koba, and Jenkins are limited by retrospective or historical comparisons or incomplete allocation reporting [21,22,24,27,28,46,48,49].

Repeated primary studies occur across the reviews, including the Landes reports, Bertossi, Spinelli, and subsequent BSSO comparisons [9,11,12,13,14,15,16]. The scoping review explicitly includes the Landes reports, Bertossi, Köhnke, Sobol, and Cascino. Review counts therefore exaggerate replication if the same participants are counted repeatedly. Design and numerical extraction should be checked against the original report: the discrepancies identified for Shirota, Köhnke, Cascino, and Sato illustrate the risk of relying on titles or secondary summaries.

Publication bias and selective reporting may favor positive device comparisons or favorable outcomes and follow-up points. Ahmadi used funnel plots and Egger and Begg tests without detecting significant asymmetry, but six studies give those procedures limited power [9]. Publication-bias analyses could not be verified for all other cited reviews. The absence of a detected signal is not evidence that reporting bias is absent. The lack of protocols or accessible full methods for several primary studies also prevents reliable assessment of omitted endpoints.

Learning requirements and costs should be treated as practical considerations. The acquisition of a generator, inserts, maintenance, sterilization, and operating-room resources contributes to cost, but higher equipment expenditure does not necessarily establish higher total procedural cost. Conversely, hypothetical savings from fewer complications or earlier discharge are not proven cost offsets. No validated cost-effectiveness conclusion can be drawn from the comparisons reviewed. Surgeon experience may affect workflow, but a direct independent learning-curve effect has not been demonstrated.

### Hybrid Instrumentation as a Research Question

Anatomical reasoning suggests that conventional instruments could be used for accessible cortical cuts and piezoelectric inserts for selected cuts close to vulnerable soft-tissue or neurovascular structures. Raffaini et al. describe a combined reciprocating-saw and piezoelectric BSSO technique [55]. This establishes technical feasibility, not comparative superiority. Existing predominantly piezoelectric groups also sometimes used chisels or saws, further complicating comparisons of supposedly exclusive modalities [21,22].

A selective hybrid workflow is therefore a plausible option to investigate, rather than the demonstrated most balanced approach. No directly comparative evidence identified in this synthesis establishes its superiority over a fully conventional or predominantly piezoelectric workflow. Prospective trials should define the steps allocated to each device, operator experience, and perioperative protocols before evaluating blood loss, sensory recovery, operative duration, complications, and cost.

## 6. Limitations and Future Research

This narrative synthesis is limited by purposive selection, incomplete access to full texts, lack of independent duplicate extraction, and absence of a formal risk-of-bias or certainty-grading exercise. Primary studies are heterogeneous in procedure, comparator, allocation, assessment, follow-up, and supplementary instruments. Missing numerical details were not estimated from qualitative statements, and a missing detail in an accessible abstract was not treated as proof that it was absent from the original study. These constraints limit both the completeness and the strength of recommendations.

Future multicenter trials should prespecify clinically relevant outcomes and separate device cutting time, osteotomy completion time, total operative duration, and operating-room time. They should report patients and operated sides separately, analyze paired observations appropriately, and document device model, inserts, irrigation, adjunctive instruments, anesthetic and medication protocols, movement magnitude, and nerve handling. Sensory testing should combine standardized objective measures and patient-reported function at fixed intervals through at least 12 months. Transfusion, major vascular injury, unfavorable splits, union, relapse, and total cost require adequate sample sizes and follow-up. Hybrid workflows warrant direct comparative testing rather than inference from technical feasibility.

## 7. Conclusions

Piezoelectric instrumentation is associated with statistically significant reductions in measured blood loss in several orthognathic comparisons, but results are not uniform and clinical importance depends on baseline risk and perioperative management. A reduction in transfusion or major vascular injury is not established. Neurosensory findings are mixed, with differences among recovery time, sensory test scores, and persistent disturbance; no precise absolute benefit can be specified. Operative-time findings depend on the interval and procedure measured, and experience has not been shown independently to explain the discrepancies.

Combined bimaxillary studies provide the most direct evidence for the complete operation; isolated osteotomy and other surgical studies have narrower applicability. Neither clinical healing superiority nor hybrid-workflow superiority has been demonstrated. Instrument choice should therefore be individualized, while stronger comparative evidence is sought for patient-important outcomes.

## Figures and Tables

**Figure 1:**
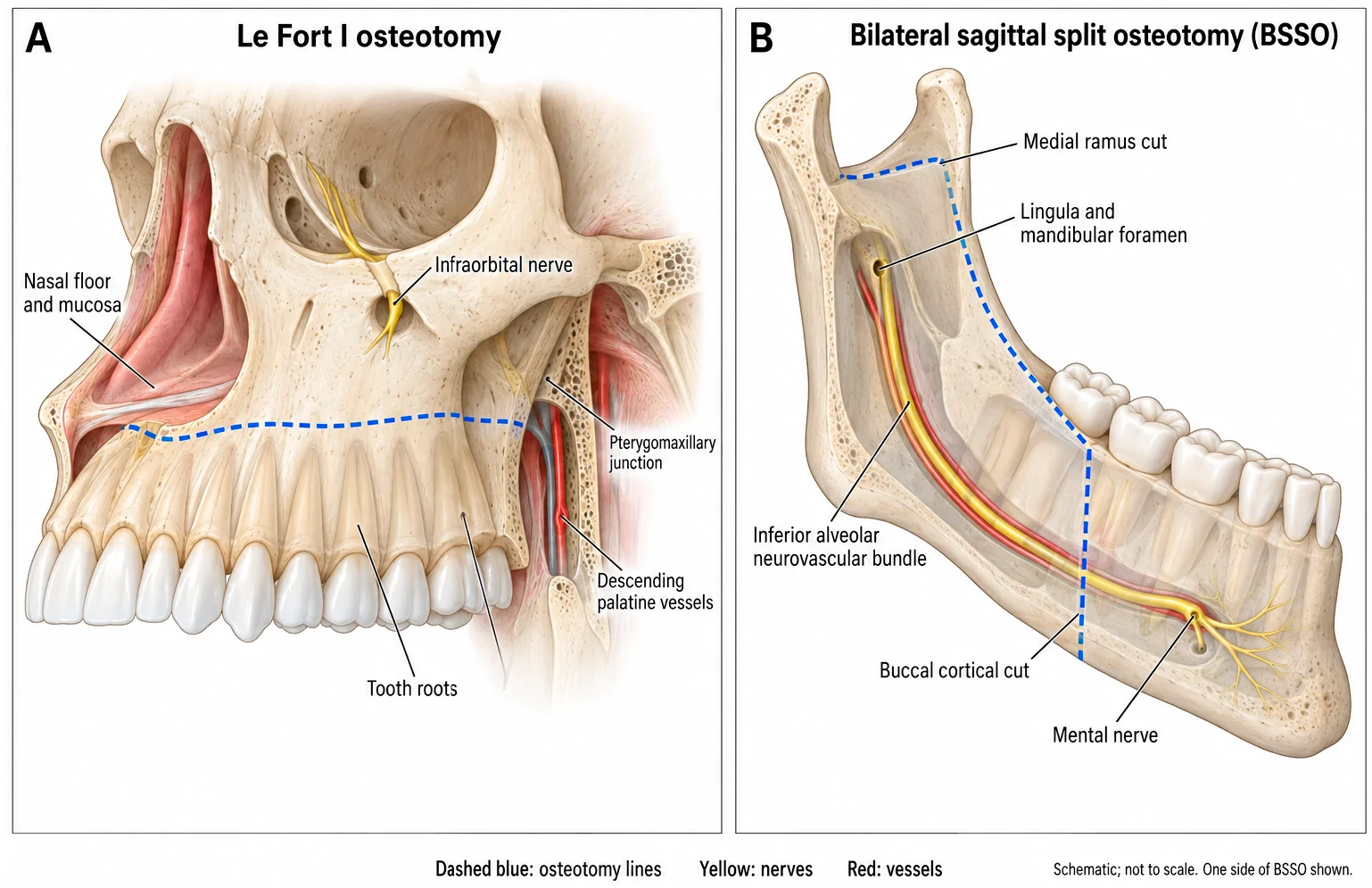
Anatomical relationships of Le Fort I osteotomy and bilateral sagittal split osteotomy (BSSO). (**A**) The maxillary osteotomy lies above the dental roots and below the infraorbital foramen; the nasal lining and descending palatine vessels are relevant adjacent structures. (**B**) One side of BSSO is shown, illustrating the medial ramus and buccal cortical cuts in relation to the lingula, mandibular foramen, inferior alveolar neurovascular bundle and mental nerve. Dashed blue lines indicate representative osteotomy paths; yellow denotes nerves and red denotes vessels. Cutaway and transparent views expose normally hidden structures. The schematic is not to scale and does not represent all technical variants or patient-specific anatomy. Illustration generated with OpenAI ImageGen 1.0.

**Table 1:** Principal comparative studies and time or blood-loss endpoints.

Study and Design	Patients Procedure and Instruments	Numerical Findings and Endpoint	Appraisal and Information Limits
Landes 2008 [21,22] Prospective piezo cohorts with conventional controls; [21] explicitly historical	50 vs. 86 [22]; 90 vs. 90 [21]. Mixed orthognathic operations; bimaxillary subsets. Piezo vs. saw/chisel; adjunctive chisels and saws used.	Bimaxillary blood loss: 541 ± 150 vs. 773 ± 344 mL, *p* = 0.001 [22]; 537 ± 208 vs. 772 ± 338 mL, *p* = 0.0001 [21]. Total operation: 227 ± 73 vs. 238 ± 61 min, *p* = 0.5; 223 ± 70 vs. 238 ± 60 min, *p* = 0.2.	Potential cohort overlap; different procedure mix and historical controls. Subset denominators and detailed paired analysis not verifiable from abstracts. Three-month sensory testing.
Spinelli 2014 [23] Within-patient comparison	12 bimaxillary patients; opposite sides treated with piezo vs. traditional saw, reversed in half of patients.	Blood loss 25% lower, *p* = 0.0367; procedure duration 35% longer, *p* = 0.0018. Absolute time, blood-loss measurement details and full follow-up schedule not verified from accessible abstract.	Small sample; side allocation not described as randomized in abstract. Paired analysis, blinding and adjunct devices not verifiable. Whole-operation and side-specific outcomes require distinction.
Bertossi 2013 [24] Reported randomized trial	110 bimaxillary patients; piezo vs. reciprocating saw/bur. Additional instruments not verified.	Reported time intervals: mandibular osteotomy on one side 3 min 31 s to 5 min 2 s vs. 7 min 23 s to 10 min 22 s; Le Fort I 5 min 17 s to 7 min 55 s vs. 8 min 38 s to 15 min 11 s. Blood loss < 300 mL vs. categories of 400 and >500 mL.	Osteotomy times are not total operation times; source wording does not establish whether intervals are ranges of individual observations. Allocation concealment, blinding, precision and baseline balance not verifiable. Sensation reported at six months.
Rossi 2018 [25] Longitudinal comparison	25 bimaxillary patients: 14 piezo and 11 saw. Device adjuncts and allocation details not verified.	Longer operation with piezo; absolute duration not available in accessible abstract. Pain difference at day 3, *p* = 0.035; swelling at day 30, *p* = 0.045.	Small unequal groups; scales, effect magnitudes, analgesic/steroid protocols and clinically important thresholds not verified. Follow-up includes day 1 and day 30.
Akbar 2017 [26] Within-patient comparative study	24 bimaxillary patients; 96 osteotomies, comprising 48 maxillary and 48 mandibular. Piezo vs. oscillating saw.	Piezo time reported as 63 min, with longer duration, *p* = 0.003. Blood loss *p* = 0.091; swelling *p* = 0.041; nerve damage *p* = 0.009.	The 63 min interval and comparator absolute time are insufficiently defined in abstract. Follow-up, paired handling, scales, adjuncts and assessor blinding not verified. Blood loss is non-significant.
Cascino 2021 [27] Retrospective in abstract methods	100 bimaxillary patients, 50 per group; same surgeon. Piezo vs. saw; supplementary instruments not verified.	Intraoperative time described as unchanged; lower analgesic use and day-2 discharge described for piezo. Exact time and analgesic values not verified.	Title says randomized, but abstract says retrospective. Allocation, baseline balance, blinding, medication and discharge protocols unresolved. Full sensory follow-up schedule not verified.
Köhnke 2017 [28] Prospective randomized split-mouth	50 patients; 100 BSSO procedures. Piezo vs. conventional mechanical osteotomy. Two specialists, each with ≥1 year piezo experience.	Incision to complete split, per side: 17 ± 6 vs. 25 ± 9 min, *p* < 0.001. Sensory testing to 12 months: no between-group difference.	Paired t/Wilcoxon and chi-square tests reported. Correlation handling for every categorical endpoint and assessor blinding not verified. BSSO timing cannot represent the complete bimaxillary operation.
Shirota 2014 [29] Retrospective non-randomized	59 mandibular setback patients: 29 piezo plus separator vs. 30 Lindemann bur plus chisel.	No independent blood-loss benefit; greater Semmes–Weinstein disturbance at 3 months with piezo, *p* = 0.008. ANCOVA included age, movement, time and fixation variables.	Comparator differs in cutting and splitting instruments. Residual confounding; blinding and detailed baseline balance not verified. Does not support a general complication reduction.
Demirbas 2020 [30] Prospective randomized single-blind comparison	34 Le Fort I patients; BoneScalpel vs. piezo device vs. Lindemann bur/reciprocating saw.	BoneScalpel shorter cutting time, *p* < 0.001, and procedure duration, *p* = 0.012; blood-loss reduction up to 45%, *p* = 0.038.	Three distinct device groups. Absolute times, group-specific follow-up and adjuncts not verified from abstract. BoneScalpel results cannot be attributed to the piezo group.
Sato and Imai 2024 [31] Sequential retrospective cohorts	22 cleft lip/palate bimaxillary patients: first 10 conventional power tools, subsequent 12 high-powered ultrasonic device.	Total surgery 223 ± 12.217 vs. 278 ± 11.153 min, *p* = 0.008. Blood loss 230 ± 45.154 vs. 343 ± 49.463 g, *p* = 0.118; normal sensation by 6 months in both groups.	Special population, chronological confounding and distinct ultrasonic device. Original units are grams. Secondary review extraction differs in units and group counts [9].

Values are piezoelectric or specified ultrasonic technique versus conventional instrumentation unless stated otherwise. ±values reproduce the original report. Details marked not verified were unavailable in the accessible original abstract or report; this is not a finding of non-reporting in the full publication. No numerical values were imputed.

**Table 2:** Principal sensory comparisons and their distinct endpoints.

Study and Unit	Assessment and Follow-Up	Result	Interpretation
Bertossi [24] 110 patients	Retained IAN sensation at 6 months; test details not verified in abstract	98.2% piezo vs. 92.7% conventional	Descriptive favorable result; CI and precise sensory denominator not verified.
Köhnke [28] 50 patients/100 sides	Two-point discrimination and static light touch; 1, 3, 6 weeks and 6, 12 months	No difference in NSD or recovery	Longer follow-up with explicit tests; no demonstrated sensory superiority.
Monnazzi [46] 20 patients/40 sides	Semmes–Weinstein; baseline, 1 and 4 weeks, 2 and 6 months; patient and examiner blinded	No statistically significant difference	Blinding strengthens assessment; small sample does not establish equivalence.
Shirota [29] 59 patients	Semmes–Weinstein at 3 months	Greater disturbance with piezo, *p* = 0.008	Non-random allocation and different splitting tools; negative finding must remain explicit.
Sobol [47] 20 patients/40 SSOs	UK MRC functional sensory recovery; followed to recovery within 1 year	Median time 94.5 vs. 101.5 days, *p* = 0.20; higher score at recovery favored piezo, HR 2.3, 95% CI 1.1–4.9, *p* = 0.04	Recovery time and degree of function differ; not evidence of faster FSR.
Koba [48] 44 patients	Paresthesia immediately and at 3 months; detailed scale not verified	No immediate difference; lower 3-month paresthesia with piezo; some persistent deficits	Retrospective setback study; comparator is conventional chisel workflow. Absolute rates not verified.
Jenkins and Langford [49] 48 patients, 24 per cohort	Neurosensory disturbance graded at 12 months; scale details not verified	Better recovery with piezo, *p* = 0.01	Single surgeon, sequential 15-month cohorts; time-period confounding possible.
Cascino [27] 100 patients	Lower-lip recovery; detailed test schedule not verified	Favorable sensory recovery described	Retrospective methods despite randomized title; no verified absolute rate or CI.

IAN, inferior alveolar nerve; NSD, neurosensory disturbance; SSO, sagittal split osteotomy; FSR, functional sensory recovery; HR, hazard ratio; CI, confidence interval. No common absolute risk reduction is derived from these heterogeneous endpoints.

**Table 3:** Main evidence syntheses and limits on pooled interpretation.

Review and Search Coverage	Evidence Base	Principal Finding	Limitations and Certainty
Silva 2017 [11] Previous 10 years; exact last date not verified	12 studies; 799 patients	≥6-month NSD 4.7% vs. 61.6%; longer ultrasonic osteotomy time	Heterogeneous studies and tests; descriptive cross-study rates are not a transferable absolute effect. Formal certainty grade not verified.
Rude 2019 [12] January 2000–November 2017	5 controlled studies	NSD ranges 1.8–23.0% vs. 7.3–52.0% at 2–12 months; no pooling	2 moderate- and 3 high-risk studies; no definitive superiority conclusion.
Pagotto 2017 [14] Last search date not verified	3 RCTs and 5 non-randomized controlled studies overall	Blood-loss MD −128 mL, *p* < 0.001; no pooled operative-time difference	Endpoint-specific counts, blood-loss CI and I^2^ not verified from abstract. Not all studies contribute to each outcome; pain/swelling not pooled.
Abd-ElHady 2022 [13] Through September 2020	6 studies; 284 participants overall	NSD at 1 week RR 0.99 (0.90–1.08), *p* = 0.79; at 3 months RR 0.39 (0.09–1.75), *p* = 0.22	High RCT risk of bias; serious/critical non-randomized risk. Outcome-specific counts and I^2^ not verified from abstract.
Alrefai 2022 [15] Last search date not verified	15 reports described: 3 conventional-only, 3 piezo-only, 9 both modalities	Blood-loss MD −140.4 mL, *p* = 0.29; NSD comparisons *p* = 0.11 at 3 months and *p* = 0.50 at 6 months	Comparative estimate denominators, CIs, I^2^ and formal certainty not verified. Favorable descriptive rates do not override non-significance.
Ahmadi 2025 [9] Through 5 October 2025	6 studies; reported 144 participants/252 procedures	WMD −81.73 mL (95% CI −97.30 to −66.16); I^2^ 51.83%	JBI appraisal, no GRADE; paired handling unclear. Counts/units differ from original Sato report [31]. Precision remains uncertain.
Navarrete 2025 [16] 2000–December 2024	9 articles; reported 731 subjects	Scoping map of heterogeneous sensory assessments and recovery	No pooled comparative effect. Primary studies recur in other reviews; maps evidence rather than establishing certainty.

**Table 4:** Outcome-level interpretation and evidential basis.

Outcome	Comparative Finding	Consistency or Evidence Type	Main Limitation
Measured blood loss	Often lower with piezo; some non-significant findings [9,14,15,21,22,23,24,25,26,27,29]	Mixed, with several significant comparisons	Protocol variation, historical controls, paired data and extraction concerns; transfusion benefit unproven.
Operative time	Longer, shorter or similar [11,14,15,21,22,23,24,25,26,27,28,49]	Inconsistent clinical evidence	Different timing intervals; independent learning-curve effect unestablished.
Neurosensory function	Positive, null and adverse findings [12,13,15,24,28,29,46,47,48,49]	Mixed clinical evidence	Tests, timing, bias and unit of analysis differ; no precise absolute benefit.
Pain and swelling	Some favorable time points, but not uniform [23,25,27,50]	Limited comparative evidence	Small samples and incompletely verified scales/protocols; patient-important effect uncertain.
Precision and selectivity	Narrow controlled cuts and relative soft-tissue selectivity [6,7,8,18,19,20,34,35]	Technical properties and feasibility	Not a surrogate for proven reduction in clinical complications.
Bone healing and stability	No established clinical difference favoring either instrument [10,52]	Insufficient comparative clinical evidence	Preclinical findings cannot establish union or relapse benefit.
Equipment and total cost	Dedicated equipment adds expenditure; total cost difference uncertain	Practical consideration	Formal comparative cost-effectiveness evidence insufficient.
Hybrid workflow	Feasible technique-combining modalities [55]	Technical report and anatomical rationale	Superiority over either complete workflow not established.

## Data Availability

No new data were created or analyzed in this study. Data sharing is not applicable to this article.
